# The role of the leukocyte glucose index in predicting clinical outcomes in acute methanol toxicity

**DOI:** 10.1016/j.toxrep.2025.101994

**Published:** 2025-03-11

**Authors:** Ola Elsayed Nafea, Walaa Gomaa Abdelhamid, Fatma Ibrahim

**Affiliations:** a Forensic Medicine and Clinical Toxicology Department, Faculty of Medicine, Zagazig University, Zagazig 44519, Egypt; b Forensic Medicine and Clinical Toxicology Department, Faculty of Medicine, Ain Shams University, Cairo 11566, Egypt

**Keywords:** In-hospital mortality, Leukocyte glucose index, Methanol, Prediction, Survival, Visual loss

## Abstract

**Introduction:**

Acute methanol poisoning signifies a global health issue. This study was designed to explore the role of the leukocyte glucose index (LGI) in predicting clinical outcomes; in-hospital mortality and visual impairment, and length of hospital stay, in acute methanol toxicity and to evaluate the association between LGI and all initial patient characteristics.

**Patients and methods:**

This was a retrospective analysis that involved 82 acutely methanol-intoxicated patients, starting from January 2021 to December 2023. Patients were categorized by on-admission LGI tertiles into low, intermediate, and high groups.

**Results:**

Approximately 27 % (22 out of 82) of patients died during hospitalization, with most of them belonging to the high LGI group. No significant differences existed in the proportions of patients with total vision loss, or the length of hospital stay. The majority of the undesirable findings were apparent in patients in either the intermediate or high LGI groups. LGI can distinguish exceptionally between survivors and non-survivors with an area under the curve of 0.808. However, LGI does not have any discriminatory power in predicting adverse visual outcomes.

**Conclusion:**

LGI can serve as a valuable tool in predicting early in-hospital mortality in acute methanol poisoning.

## Highlights

• LGI can serve as a valuable prognostic tool in acute methanol poisoning.
• LGI can distinguish between survivors and non-survivors with an AUC of 0.808.
• LGI does not have any discriminatory power in predicting adverse visual outcomes.

## Graphical Abstract

**Figure:**
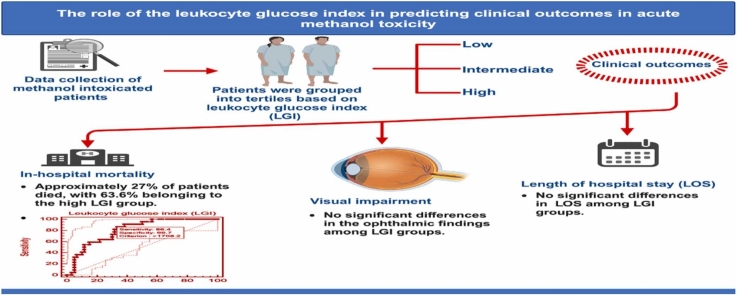

## 1 Introduction

Acute methanol poisoning signifies a global health issue, particularly in the developing world [1]. Acute methanol poisoning necessities timely proper intervention to prevent severe disability and death. Intake of homemade alcoholic beverages is a common source of poisoning especially in countries that legally prohibit alcoholic beverages [2], [3]. In addition, adulterated alcoholic beverages are legally sold alcohol products that have been illegally altered [4]. Unauthorized workrooms, along with products that are re-packaged in the packaging of well-known brands or smuggled and illegally, even if genuine, pose concealed risks of methanol exposure [5]. False beliefs about methanol's potential to prevent viral infections have emerged during the COVID-19 pandemic, driving people to ingest methanol [6], [7].

Methanol itself has a minimum intrinsic toxicity; however, it is metabolized *in vivo* into two highly toxic metabolites, formic acid, and formaldehyde, which block the cytochrome oxidase system in the mitochondria, suppressing oxidative phosphorylation, with a subsequent deficiency of adenosine triphosphate to which brain and retina are particularly vulnerable. When toxic metabolites accumulate, disruption of cellular respiration takes place, leading to prominent tissue hypoxia and metabolic acidosis [8]. Other pathological events include an interaction of toxic metabolites and a decrease in energy supply which cause a disruption of biological oxidation and the development of apoptotic cell death in the optic nerve, and necrotic injury in the basal ganglia and subcortical white matter [8], [9].

Clinical findings of methanol poisoning include visual impairment, difficult breathing, nausea, abdominal pain, hypoxia, and acid-base imbalance. Moreover, permanent vision loss can develop in a large proportion of victims secondary to optic nerve atrophy. Long-term sequalae like executive and memory deficits are reported in the surviving patients. Any delay in initiation of treatment results in death and disability [8], [9], [10], [11], [12]. According to the American Academy of Clinical Toxicology Guidelines for methanol poisoning, treatment modalities include reversal of metabolic acidosis with sodium bicarbonate, extracorporeal elimination to hasten the excretion of methanol and formic acid and to aid in metabolic acidosis correction, along with limitation of methanol breakdown with an alcohol dehydrogenase inhibitor, e.g., ethanol or fomepizole, and folic acid to enhance the metabolism of formic acid [13].

Hyperglycemia is a frequent complication in critically ill patients. A significant association between on-admission blood glucose level and patient outcomes has been widely documented [14], [15], [16]. In a diversity of life-threatening poisons, such as aluminum phosphide, organophosphorus pesticides, and methanol, elevated blood glucose level on presentation is independently associated with lethality [17], [18], [19]. This could be clarified by stress-induced hyperglycemia. The acute stress of methanol poisoning may contribute to hyperglycemia. Stress-induced hyperglycemia leads to a condition of insulin insensitivity and elevated blood glucose by various pathogeneses.

Stress-induced hyperglycemia leads to a state of insulin resistance and elevated blood glucose levels through several mechanisms. The intensified action of the insulin counterregulatory hormones, such as adrenaline, cortisol, glucagon, and growth hormone, disrupts glucose homeostasis. In addition, the upregulation of inflammatory cytokines also contributes to this disturbance in glucoregulation. Furthermore, the failure of skeletal muscle to uptake glucose via the glucose transporter type 4 is another mechanism involved [20], [21].

Although accumulation of toxic metabolites and tissue hypoxia are the fundamental mechanisms of methanol poisoning, other proposed mechanisms of methanol toxicity include inflammation, lipid peroxidation, and oxidant injury [22], [23], [24].

Leukocyte glucose index (LGI) is a novel biomarker derived from blood leukocytes and glucose values. Recently, the potential of LGI to predict the prognosis of diverse cardiac diseases has been extensively studied [25]. Elevated LGI has been linked with poor outcomes in acute myocardial infarction patients [26], [27] as well as with the severity of coronary artery disease [28], [29] and COVID-19 infection [30].

Notably, no study, so far has explored whether LGI has a prognostic role in patients with acute methanol toxicity. Against this background, this study was implemented to explore the role of LGI in predicting clinical outcomes (in-hospital mortality and visual impairment, as well as length of hospital stay) in acute methanol toxicity and to evaluate the association between LGI and all initial patient characteristics. Enhancing risk stratification, optimizing treatment strategies, and ultimately improving outcomes in acute methanol poisoning are critical in patient management.

## 2 Patients and methods

### 2.1 Study design and setting

This was a retrospective analysis that involved gathering the necessary information from the patient medical records admitted to Zagazig University Poisoning Treatment Unit, Zagazig University Hospitals, Zagazig, Egypt, starting from January 2021 to December 2023. Data confidentiality was maintained and used only for epidemiologic purposes. The Institutional Review Board of the Faculty of Medicine, Zagazig University approved the study protocol (ZU-IRB # 382-June-2024). As a retrospective study, the need for individual patient consent was waived.

### 2.2 Eligibility criteria

This study included 82 patients with acute methanol poisoning who were aged 18 years and older. Patients meeting the following criteria were typically excluded from the study: diagnosis of malignancy, known hematological disorders, history of blood transfusion in the past three months, history of co-ingestants or exposure to other toxins, history of ocular disorders, history of systemic diseases (including diabetes mellitus, cardiopulmonary, renal, and hepatic diseases), pregnancy and lactation, receipt of chemotherapy or radiation therapy, and recent infections in the past two weeks. Diagnosis of methanol poisoning was established by history of methanol exposure, physical examination, toxicology, and laboratory assessment according to the Zagazig University Poisoning Treatment Unit protocols.

### 2.3 Data collection

Data collected included patient initial characteristics: age, gender, residence, history of substance abuse, presence of psychiatric illness, intent of poisoning, time from poisoning to hospital admission (delay time), initial clinical assessment (level of consciousness, vital signs, electrocardiogram (ECG) findings, and ophthalmic assessment) at time of admission. In addition, initial arterial blood gas analysis and laboratory findings including blood methanol level, serum urea and creatinine, hepatic aminotransferases, creatine phosphokinase (CPK), blood glucose, hematological indices, hemoglobin (HB), hematocrit, total leukocytic count (TLC), platelet count, and red cell distribution width (RDW). Additionally, patient outcomes including in-hospital mortality, total vision loss, and total length of hospital stay were also recorded.

Calculation of LGI was done using the following formula according to León Aliz [31].

LGI= [blood glucose (mg/dL) × TLC (cells/mm^3^)/1000]. *Blood glucose level and TLC had been measured at the time of admission.*

### 2.4 A post hoc power calculation

The power of the current study is 97.1 %. Because the study is retrospective, a post hoc power was calculated by G*power 3.1 [32] based on (Means: Mann–Whitney *U* test), mean±SD of LGI values in survivors (1639.59 ± 1060.33 mg/dL.mm^3^) versus non-survivors (2707.79 ± 1082.88 mg/dL.mm^3^) and at a sample size of 82 (60 survivors vs 22 non-survivors) and a type I error threshold (α) < 0.05.

### 2.5 Statistical analysis

Continuous variables were presented as median with 25th-75th percentiles or mean ± standard deviation (SD) according to the normality of distribution which was tested by the Shapiro-Wilk test. Categorical variables were presented as count and proportions. A Kaplan-Meier survival plot was used to illustrate the probability of patient survival during hospital admission. The receiver operating characteristic (ROC) curve was employed to evaluate the prognostic performance of the LGI in predicting clinical outcomes. The optimal cut-off points for LGI were determined using Youden's index, which is calculated as (sensitivity + specificity - 1), and the maximized area under the curve (AUC). Differences were considered statistically significant at a *P*-value ≤ 0.05. The Statistical Package for the Social Sciences (SPSS®) STATISTICS software, Version 26.0.0.0 (IBM Corporation, Armonk, New York, USA) was used for all statistical analyses. All statistical comparisons were two-tailed.

## 3 Results

**Fig. 1:**
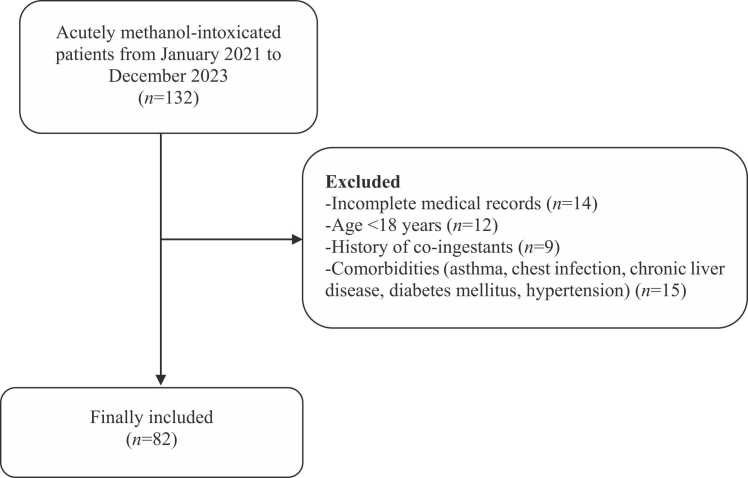
Study flowchart.

**Fig. 2:**
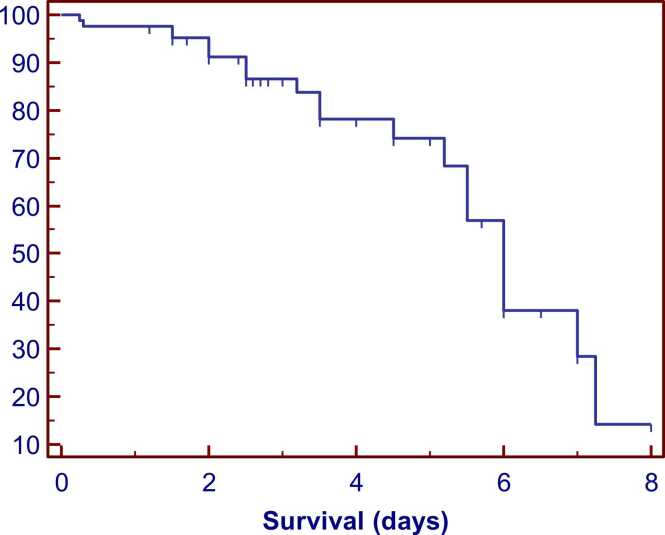
Kaplan-Meier survival curve showing the probability of methanol-intoxicated patients’ survival during hospital admission.

**Fig. 3:**
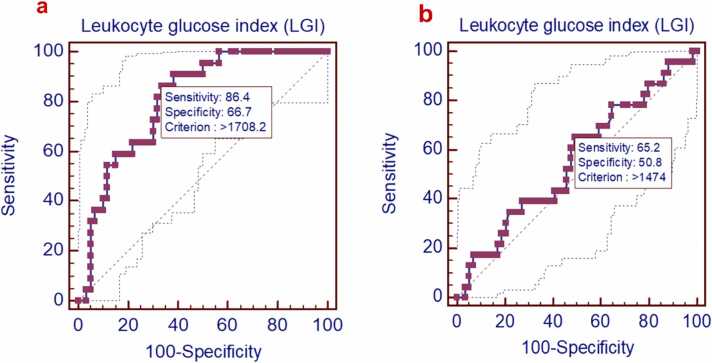
The receiver operating characteristic (ROC) curves for on admission LGI in the prediction of clinical outcomes of methanol-intoxicated patients. Panel a: ROC curves for in-hospital mortality. Panel b: ROC curves for total vision loss.

**Fig. 4:**
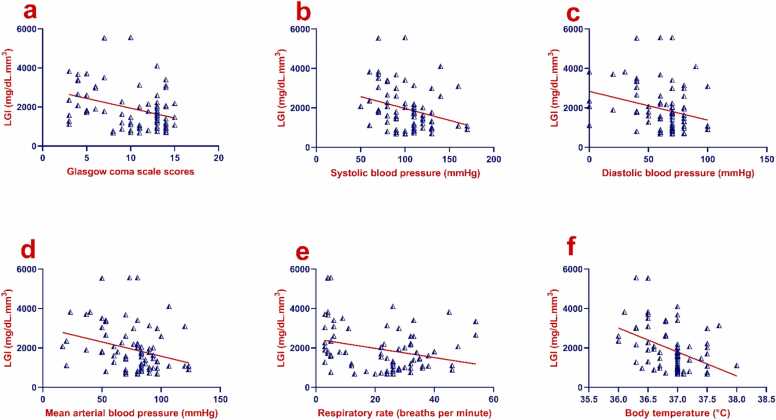
a-f. Scatter plots with regression lines of on-admission LGI and initial clinical findings of methanol-intoxicated patient characteristics. Abbreviation: LGI, Leukocyte glucose index.

**Fig. 5:**
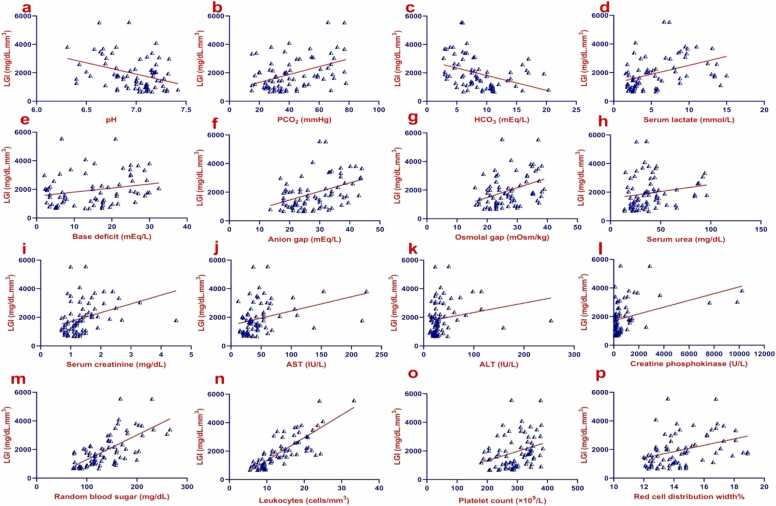
a-p. Scatter plots with regression lines of on-admission LGI, arterial blood gas and initial laboratory findings of methanol-intoxicated patients. Abbreviations: ALT, Alanine aminotransferase; AST, Aspartate aminotransferase; HCO3, Bicarbonate; LGI, Leukocyte glucose index; PCO2, Partial pressure of carbon dioxide.

Fig. 1 illustrates the study flowchart. All patient initial characteristics and in-hospital management measures were categorized by on-admission LGI tertiles into low, intermediate, and high groups. Among the 82 eligible records, the mean age was 34.8 years (SD ±8.3). Men represented approximately 96 % of the cases, and intentional poisoning accounted for about 91 %. All cases were exposed to methanol through ingestion. Patients in the high LGI group had greater proportions of substance abuse history (*P* = 0.031), lower Glasgow coma scale scores (*P* = 0.009), and abnormal ECG findings (*P* = 0.026). In addition, systolic (*P* = 0.020), diastolic (*P* = 0.028), and mean arterial blood pressures (*P* = 0.017) were significantly lower in the high LGI group compared to the low LGI group. While heart rate was significantly lower in both intermediate (*P* = 0.029) and high (*P* = 0.027) LGI groups compared to the low LGI group. Body temperature was significantly lower in the high LGI group than the intermediate (*P* = 0.013) and low LGI (*P* = 0.001). Other baseline patient characteristics are demonstrated in Table 1. Table 2 shows that all ophthalmic findings were not significantly different among patients categorized according to LGI tertiles (*P* > 0.05). Compared to the low LGI group, the high LGI group had significantly lower pH (*P* = 0.006), and arterial bicarbonate (*P* < 0.001) but significantly higher PCO_2_ (*P* = 0.002), arterial lactate (*P* < 0.001), osmolal gap (*P* = 0.002), ALT activity (*P* = 0.004), platelet count (*P* = 0.01), and RDW(*P* < 0.001). Anion gap, serum creatinine level, and AST activity were significantly elevated in the high LGI group compared to both intermediate and low LGI groups [(*P* = 0.024 and *P* < 0.001), (*P* = 0.004 and *P* < 0.001), and (*P* = 0.033 and *P* < 0.001), respectively]. Both blood glucose level and TLC were significantly elevated in the intermediate and high LGI groups compared to the low LGI group [(*P* = 0.007 and *P* < 0.001), and (*P* = 0.002 and *P* < 0.001), respectively], additionally, the high LGI group had significantly higher levels of blood glucose level (*P* < 0.001), and TLC (*P* = 0.001) than the intermediate LGI group as demonstrated in Table 3. Regarding the clinical outcomes, Fig. 2 shows the probability of survival during hospitalization for methanol-intoxicated patients, with a median survival time of 6 days and a 95 % confidence interval (CI) of 5.42–6.58. Approximately 27 % (22 out of 82) of patients died during hospitalization. Of the 22 patients who died, around 64 % (14 out of 22) belonged to the high LGI group. The proportions of patients with total vision loss (*P* = 0.70) and the length of hospital stay (*P* = 0.14) were not significantly different among LGI groups, as shown in Table 4. Regarding the performance of on-admission LGI for predicting the clinical outcomes among cases, the ROC curve analysis revealed that LGI can distinguish between survivors and non-survivors with an area under the curve (AUC) of 0.808, an excellent discriminative power [(95 % (CI): 0.706–0.886, (*P* < 0.001)] (Table 5 and Fig. 3a). The optimal sensitivity and specificity were 86.4 % and 66.7 %, respectively at a cutoff value> 1708.2 mg/dL.mm^3^. While LGI demonstrated a poor performance in the prediction of total vision loss, with an AUC of 0.557, (*P* = 0.43), no discriminative power (Table 5 and Fig. 3b). Table 6, Fig. 4, Fig. 5 shows correlations between on-admission LGI and initial methanol-intoxicated patient characteristics. Moreover, LGI was inversely correlated with Glasgow coma scale, systolic blood pressure, diastolic blood pressure, mean arterial blood pressure, respiratory rate, body temperature, pH, arterial bicarbonate and positively correlated with PCO_2_, arterial lactate, base deficit, anion gap, osmolal gap, urea, creatinine, AST, ALT, CPK, blood glucose level, TLC, platelet count, and RDW (*P* < 0.05).

**Table 1:** Baseline characteristics of methanol-intoxicated patients categorized by LGI on-admission.

	LGI (mg/dL.mm^3^)				
	All patients	Low	Intermediate	High	*P*-value
		< = 1199	1199.01–2068	≥ 2068.01	
	*n* = 82	*n* = 27	*n* = 27	*n* = 28	
Age (years), (mean ± SD)	34.8 ± 8.3	33.7 ± 9.7	33.4 ± 6.9	37.4 ± 7.8	0.14 ☆
Male gender, *n*(%)	79(96)	27(100)	24(89)	28(100)	0.066 †
Urban residence, *n*(%)	60(73)	22(82)	19(70)	19(68)	0.48 ‡
History of substance abuse ¶, *n*(%)	45(55)	12(44)	12(44)	21(75)	**0.031** ‡
History of psychiatric illness, *n*(%)	13(16)	6(22)	2(7)	5(18)	0.36 †
Intention of poisoning, *n*(%)					
Unknown	3(4)	0(0)	1(4)	2(7)	0.86 †
Unintentional	4(5)	1(4)	2(7)	1(4)	
Intentional	75(91)	26(96)	24(89)	25(89)	
Glasgow coma scale, *n*(%)					
13–15	34(42)	11(41)	14(52)	9(32)	**0.009** †
9–12	24(29)	13(48)	6(22)	5(18)	
3–8	24(29)	3(11.1)	7(26)	14(50)	
Delay time (hr)	16(10−24)	19(10−25)	15(10−24)	15.5(10–25.5)	0.83 §
Vital signs					
Systolic blood pressure (mmHg)	105(88−120)	110(90−130)	110(90−120)	90(70−110)^a^	**0.020** §
Diastolic blood pressure (mmHg)	70(58−73)	70(60−80)	70(60−70)	60(40−70)^a^	**0.028** §
Mean arterial blood pressure (mmHg)	80(66−90)	83(70−93)	83(70−90)	70(50−80)^a^	**0.017** §
Heart rate (bpm)	90 ± 24.1	100 ± 20.2	86 ± 22.9^a^	85 ± 26.7^a^	**0.04** ☆
Respiratory rate (breaths per minute)	24(8−32)	26(20−32)	24(8−32)	10(4−32)	0.11 §
Body temperature (°C)	37(36.6–37)	37(37–37.1)	37(37−37)	36.6(36.3–37)^ab^	**0.001** §
Abnormal ECG finding ∥, *n*(%)	48(59)	12(44)	14(52)	22(79)	**0.026** ‡

Bold values denote statistically significant differences at *P* ≤ 0.05 level.

Data are median (25th to 75th percentiles), unless otherwise mentioned.

☆ Ordinary one-way analysis of variance (ANOVA).

† Fisher’s exact test (R×C).

‡ Chi-squared test.

§ Kruskal-Wallis *H* test.

¶ Substance abuse includes ethanol, cannabis, tobacco, and opiates.

∥ Abnormal ECG findings include: sinus tachycardia, prolonged QTc interval, right bundle branch block, and nonspecific T-wave changes.

^a^ significant vs low LGI, ^b^ significant vs intermediate LGI by least significant difference post hoc test following ordinary ANOVA test or Dunn’s post hoc test following Kruskal-Wallis *H* test.

Abbreviations: ECG, electrocardiogram; LGI, Leukocyte glucose index.

**Table 2:** Ophthalmic assessment of methanol-intoxicated patients categorized by on-admission LGI.

	LGI (mg/dL.mm^3^)				
	All patients	Low	Intermediate	High	*P*-value
		< = 1199	1199.01–2068	≥ 2068.01	
	*n* = 82	*n* = 27	*n* = 27	*n* = 28	
Vision, *n*(%)					
Normal	14(17)	8(30)	1(4)	5(18)	0.11 ☆
Decrease visual acuity	31(38)	7(26)	12(44)	12(43)	
Diplopia	14(17)	6(22)	6(22)	2(7)	
Total vision loss	23(28)	6(22)	8(30)	9(32)	
Pupillary response, *n*(%)					0.66 †
Normal	23(28)	14(52)	9(33)	10(36)	
Sluggish	26(32)	7(26)	10(37)	9(32)	
Dilated fixed	23(28)	6(22)	8(30)	9(32)	
Retina *n*(%)					0.53 ☆
Normal	70(85)	23(85)	22(82)	25(89)	
Edema	8(10)	3(11)	2(7)	3(11)	
Bleeding	4(5)	1(4)	3(11)	0(0)	
Optic disc, *n*(%)					0.52 ☆
Normal	41(50)	15(56)	10(37)	16(57)	
Edema	13(16)	4(15)	5(19)	4(14)	
Pallor	17(21)	3(11)	8(30)	6(21)	
Hemorrhage	11(13)	5(19)	4(15)	2(7)	
Optic nerve, *n*(%)					0.70 †
Normal	59(72)	21(78)	19(70)	19(68)	
Atrophy	23(28)	6(22)	8(30)	9(32)	

☆ Fisher’s exact test (R×C).

† Chi-squared test.

Abbreviation: LGI, Leukocyte glucose index.

**Table 3:** Initial arterial blood gas and laboratory findings of methanol-intoxicated patients categorized by on admission LGI.

	LGI (mg/dL.mm^3^)				
	All patients	Low	Intermediate	High	*P*-value
		< = 1199	1199.01–2068	≥ 2068.01	
	*n* = 82	*n* = 27	*n* = 27	*n* = 28	
pH	6.99 ± 0.25	7.09 ± 0.17	6.98 ± 0.25	6.099 ± 0.28^a^	**0.005** ☆
PCO_2_ (mmHg)	41.77 ± 17.58	33.52 ± 11.81	41.93 ± 17	49.57 ± 19.59^a^	**0.002** ☆
HCO_3_ (mEq/L)	8.4(6−11)	10.8(9.3–11)	8.2(6.1–10.7)	6(4.6–8)^a^	**< 0.001** †
Arterial lactate (mmol/L)	3.9(2.5–7.63)	2.7(2.3–3.9)	3.4(2.5–8.8)	7.15(4.28–9.5)^a^	**< 0.001** †
Base deficit (mEq/L)	14.65(5.7–21.38)	6.9(5.2–17.4)	15.6(6.8–21.3)	18.15(6.7–25)	0.055 †
Anion gap (mEq/L)	28 ± 7.89	24.53 ± 6.05	27.37 ± 7.51	31.93 ± 8.31^ab^	**0.001** ‡
Osmolal gap (mOsm/kg)	26.61 ± 6.28	23.65 ± 4.75	26.87 ± 6.23	29.22 ± 6.59^a^	**0.002** ☆
Blood methanol level (mg/dL)	120(70−180)	127(80−195)	85(55−150)	143(75–174.3)	0.27 †
Urea (mg/dL)	35(24−45)	32(22−38)	36(24−46)	40(27.5–25.8)	0.062 †
Creatinine (mg/dL)	1.2(0.9–1.5)	1.1(0.8–1.3)	1.1(0.9–1.4)	1.55(1.3–1.88)^ab^	**< 0.001** †
AST (IU/L)	37(28–54.3)	28(21−43)	36(32−44)	54.5(38.3–77.5)^ab^	**< 0.001** †
ALT (IU/L)	26(23–39.3)	24(20−27)	28(23−30)	37(24–59.5)^a^	**0.004** †
CPK (U/L)	362(202.8–869)	387(205−503)	235(156–1207)^a^	547(320.5–1543.8)	**0.012** †
RBS (mg/dL)	137.49 ± 42.82	107 ± 24.48	133.85 ± 36.06^a^	170.39 ± 43.2^ab^	**< 0.001** ‡
HB (g/dL)	15.5 ± 1.86	15.42 ± 1.61	15.69 ± 2.02	15.4 ± 1.99	0.82 ‡
Hematocrit%	46.84 ± 5.73	44.92 ± 5.49	48.19 ± 5.47	47.38 ± 5.90	0.090 ‡
TLC (cells/mm^3^)	11.7(9.28–17.63)	8.9(7.5–9.4)	11.3(9.3–15)^a^	18.4(15.3–21.8)^ab^	**< 0.001** †
Platelet count (×10^9^/L)	293.71 ± 52.49	280 ± 40.17	283.54 ± 60.43	316.54 ± 48.67^a^	**0.011** ☆
RDW%	14.37 ± 1.81	13.58 ± 1.08	14.28 ± 2.17	15.22 ± 1.65^a^	**< 0.001** ☆

Bold values denote statistically significant differences at the *P* ≤ 0.05 level.

Data are median (25th to 75th percentiles) or mean±SD

☆ Welch one-way analysis of variance (ANOVA).

† Kruskal-Wallis H test.

‡ Ordinary one-way analysis of variance (ANOVA).

^a^ significant vs low LGI, ^b^ significant vs intermediate LGI by Games-Howell post hoc test following Welch ANOVA test, Dunn’s post hoc test following Kruskal-Wallis *H* test or least significant difference post hoc test following ordinary ANOVA test.

Abbreviations: ALT, Alanine aminotransferase; AST, Aspartate aminotransferase; CPK, Creatine phosphokinase; HB, Hemoglobin; HCO_3_, Bicarbonate; LGI, Leukocyte glucose index; PCO_2_, Partial pressure of carbon dioxide; RBS, Random blood sugar; RDW, Red cell distribution width; TLC, Total leukocytic count.

**Table 4:** Clinical outcomes of methanol-intoxicated patients categorized by on-admission LGI.

	LGI (mg/dL.mm^3^)				
	All patients	Low	Intermediate	High	*P*-value
		< = 1199	1199.01–2068	≥ 2068.01	
	*n* = 82	*n* = 27	*n* = 27	*n* = 28	
In-hospital mortality, *n*(%)	22(27)	1(4)	7(26)	14(50)	**0.001** ☆
Total vision loss, *n*(%)	23(28)	6(22)	8(30)	9(32)	0.70 ☆
Total length of hospital stay (days)	3(2.3–4)	3(2.5–3.5)	2.5(2−4)	3.8(2.5–5.4)	0.14 †

Bold values denote statistically significant differences at the *P* ≤ 0.001 level.

Data are median (25th to 75th percentiles), unless otherwise mentioned.

☆ Chi-squared test.

†Kruskal-Wallis *H* test.

Abbreviation: LGI, Leukocyte glucose index.

**Table 5:** The performance of on-admission LGI for predicting the clinical outcomes in methanol-intoxicated patients.

LGI (mg/dL.mm^3^)	In-hospital mortality	Total vision loss
Sensitivity% (95 % CI)	86.4(65.1–97.1)	65.2(42.7–83.6)
Specificity% (95 % CI)	66.7(53.3–78.3)	50.9(37.5–64.1)
Youden index	0.531	0.161
*C* Statistic/AUC (95 % CI)	0.808(0.706–0.886)	0.557(0.443–0.667)
*P-*value	**< 0.001**	0.43

Bold value denotes statistically significant differences at the *P* ≤ 0.001 level.

Abbreviations: AUC, Area under the curve; CI, Confidence interval; LGI, Leukocyte glucose index.

**Table 6:** Correlations between on-admission LGI and initial methanol-intoxicated patient characteristics.

Initial patient characteristics	LGI (mg/dL.mm^3^)	*P*-value
	Correlation coefficient	
Age (years)	*r* = 0.174	0.12
Glasgow coma scale, *n*(%)	*r*_*s*_= −0.288	**0.009**
Delay time (hr)	*r*_*s*_= −0.14	0.90
Systolic blood pressure (mmHg)	*r*_*s*_= −0.288	**0.009**
Diastolic blood pressure (mmHg)	*r*_*s*_= −0.278	**0.011**
Mean arterial blood pressure (mmHg)	*r*_*s*_= −0.290	**0.008**
Heart rate (bpm)	*r*_*s*_= −0.189	0.089
Respiratory rate (breaths per minute)	*r*_*s*_= −0.243	**0.028**
Body temperature (°C)	*r*_*s*_= −0.409	**< 0.001**
pH	*r*_*s*_= −0.307	**0.005**
PCO_2_ (mmHg)	*r*_*s*_= 0.369	**0.001**
HCO_3_ (mEq/L)	*r*_*s*_= −0.481	**< 0.001**
Arterial lactate (mmol/L)	*r*_*s*_= 0.468	**< 0.001**
Base deficit (mEq/L)	*r*_*s*_= 0.258	**0.019**
Anion gap (mEq/L)	*r*_*s*_= 0.404	**< 0.001**
Osmolal gap (mOsm/kg)	*r*_*s*_= 0.388	**< 0.001**
Blood methanol level (mg/dL)	*r*_*s*_= 0.032	0.78
Urea (mg/dL)	*r*_*s*_= 0.282	**0.010**
Creatinine (mg/dL)	*r*_*s*_= 0.483	**< 0.001**
AST (IU/L)	*r*_*s*_= 0.456	**< 0.001**
ALT (IU/L)	*r*_*s*_= 0.335	**0.002**
CPK (U/L)	*r*_*s*_= 0.331	**0.002**
RBS (mg/dL)	*r*_*s*_= 0.670	**< 0.001**
HB (g/dL)	*r* = 0.128	0.25
Hematocrit%	*r*_*s*_= 0.202	0.069
TLC (cells/mm^3^)	*r*_*s*_= 0.827	**< 0.001**
Platelet count (×10^9^/L)	*r* = 0.294	**0.007**
RDW%	*r*_*s*_= 0.417	**< 0.001**

Bold values denote statistically significant differences at the *P* ≤ 0.05 level.

Total number= 82

Abbreviations: ABG, Arterial blood gas, ALT, Alanine aminotransferase; AST, Aspartate aminotransferase; CPK, Creatine phosphokinase; HB, Hemoglobin; HCO_3_, Bicarbonate; LGI, Leukocyte glucose index; PCO_2_, Partial pressure of carbon dioxide; RBS, Random blood sugar; *r*, Pearson's correlation coefficient; RDW, Red cell distribution width; *r*_s_, Spearman’s rank correlation coefficient.

## 4 Discussion

The current findings demonstrate the potential of LGI in predicting in-hospital mortality in acute methanol poisoning. However, LGI does not have any discriminatory power in predicting adverse visual outcomes in these patients.

A previous retrospective study of Sanaei-Zadeh et al. [17] concluded that blood glucose levels were significantly higher in non-survivors than survivors following methanol intoxication as well as elevated blood glucose level heralds for a greater risk of mortality following acute methanol poisoning. Methanol poisoning in a diabetic patient was accompanied by diabetic ketoacidosis. This condition highlights the hyperglycemic effect of methanol [33]. The current findings demonstrated that LGI was positively correlated with blood glucose on admission. Patients with LGI level ≥ 2068.01 mg/dL.mm^3^ had the highest blood glucose compared to other patients.

Clinical studies have shown that inflammatory biomarkers derived from blood cell counts can predict poor outcomes, such as mortality and severity, in cases of acute poisoning, e.g., carbon monoxide, methanol, glufosinate ammonium, methanol, mushroom, and pesticides. Specifically, these biomarkers are based on the levels of neutrophils, monocytes, platelets, and lymphocytes in the blood. Former studies indicate that monitoring these inflammatory parameters can provide valuable prognostic information regarding the course and severity of acute poisoning cases [34], [35], [36], [37], [38], [39], [40].

We found that LGI was positively correlated with white blood cells at presentation. Patients with LGI level ≥ 2068.01 mg/dL.mm^3^ had the highest white blood cells compared to others. In support, in a mouse model of acute methanol poisoning, an overproduction of serum proinflammatory cytokines (interleukin-2, interferon-γ, and tumor necrosis factor-α) was witnessed in response to staphylococcal enterotoxin B. This in turn, leads to further activation of the immune system and recruitment of leukocytes. Recruitment of leukocytes to the site of lesion is a primary event in the control of an inflammatory process [41], [42]. Leukocytosis can be an indicator of the severity of methanol poisoning. Higher leukocyte counts may correlate with more severe poisoning and greater tissue damage [43].

We found that LGI was inversely correlated with Glasgow coma scale and patients with LGI level ≥ 2068.01 mg/dL.mm^3^ had greater proportions of lower Glasgow coma scale scores. In support, disturbed consciousness level at time of patient presentation is associated with unfavorable clinical outcomes following acute methanol intoxication [43], [44], [45].

High anion gap metabolic acidosis is a hallmark of methanol poisoning. The development of metabolic acidosis is multifactorial, but formic acid accumulation is the prime mechanism [46].

In the current work, on-admission LGI was inversely correlated with pH and bicarbonate and was positively correlated with PCO_2_, lactate, base deficit, anion gap, and osmolal gap. Moreover, patients in the intermediate and high LGI groups had the worst arterial blood gas analysis findings. While patients with LGI level ≥ 2068.01 mg/dL.mm^3^ had the highest anion gap. Several studies correlate the severity of methanol poisoning to the degree of metabolic acidosis [44], [47], [48].

In support to the present findings, victims during methanol poisoning outbreak who had low Glasgow coma scale scores, hypotension, hyperglycemia, metabolic acidosis, and high lactic acid levels at presentation experienced worse prognosis [49].

RDW is a numerical indicator of variation in the size or volume of erythrocytes and is routinely a part of a complete blood count. It has been reported that elevated RDW values portended a greater risk of mortality and morbidity in several toxins like carbon monoxide, methanol, and pesticides [50], [51], [52]. Our findings revealed that LGI was positively correlated with RDW, additionally, patients having high LGI level had significantly higher RDW than those with low LGI level.

The present results revealed that patients in the high LGI group had lower body temperature and higher serum creatinine, moreover, on-admission LGI was inversely correlated with body temperature and positively correlated with serum creatinine. Lee and coworkers [45] reported that hypothermia and elevated serum creatinine level signified major risk factors associated with methanol mortality.

A 2023 meta-analysis of 11 studies involving 3701 patients assessed the predictive role of the LGI in the prognosis of acute myocardial infarction. The results verified the role of LGI in predicting the in-hospital mortality and acute major cardiac complications following myocardial infarction [27].

The current study had several limitations. First, there were incomplete data in patients’ files such as clinical, laboratory, or radiographic evidence of acute pancreatitis, which can clearly affect the blood glucose level as well as comprehensive neurological assessment is also lacking. Second, as a retrospective study, the potential for confounding could not be ruled out. Third, brain imaging (such as CT or MRI scans) results were unavailable for the patients with low Glasgow coma scale scores in our study. Fourth, this was a single-center study with a relatively small sample size, which may limit the generalizability of the findings, although the study has excellent post hoc statistical power.

## 5 Conclusion

LGI can serve as a valuable tool in predicting early in-hospital mortality in acute methanol poisoning. Leveraging the LGI as a prognostic tool can enhance patient risk stratification, aid in clinical decision making, facilitate proper patient management, and eventually improve patient prognosis.

## Funding

The authors confirm they did not receive any funding to implement this work.

## CRediT authorship contribution statement

**Abdelhamid Walaa:** Writing – review & editing, Writing – original draft, Conceptualization. **Ibrahim Fatma:** Writing – review & editing, Methodology, Investigation. **Nafea Ola:** Writing – review & editing, Writing – original draft, Software, Formal analysis, Data curation, Conceptualization.

## Declaration of Competing Interest

The authors declare that they have no known competing financial interests or personal relationships that could have appeared to influence the work reported in this paper.

## Data Availability

Data will be made available on request.
